# Persistent Socioeconomic Concentration of Cesarean Delivery in Peru Across Selected Survey-Based Obstetric Marker Strata: A Serial Cross-Sectional Analysis of ENDES Rounds, 2018–2024

**DOI:** 10.3390/healthcare14193263

**Published:** 2026-10-02

**Authors:** Víctor Juan Vera-Ponce, Fiorella E. Zuzunaga-Montoya, Marcos Daniel García-Rodríguez, Witre Omar Padilla, José Celso Paredes Carranza

**Affiliations:** Facultad de Medicina (FAMED), Universidad Nacional Toribio Rodríguez de Mendoza de Amazonas (UNTRM), Chachapoyas 01001, Peru; fiorella.zuzunaga@untrm.edu.pe (F.E.Z.-M.); marcos.garcia@untrm.edu.pe (M.D.G.-R.); witre.padilla@untrm.edu.pe (W.O.P.); jose.paredes@untrm.edu.pe (J.C.P.C.)

**Keywords:** cesarean section, socioeconomic inequalities in health, concentration index, health surveys, complex survey design, maternal health, Peru

## Abstract

**Background/Objectives:** Cesarean delivery is essential when clinically indicated, but socioeconomic differences in utilization may reflect differences in access, care pathways, preferences, provider practice, or facility characteristics. This study estimated survey-round-specific weighted prevalence of cesarean delivery by household wealth in Peru, quantified socioeconomic inequality, and examined whether the gradient persisted after sequential adjustment and across selected survey-based obstetric marker strata. **Methods:** We conducted a serial cross-sectional analysis of ENDES (Encuesta Nacional Demográfica y de Salud Familiar) rounds 2018–2024 using each woman’s most recent live birth occurring 0–59 months before interview. The official annual REC0111, REC21, and REC41 modules were linked using audited woman- and birth-level keys. Survey-round-specific analyses used original sampling weights; pooled analyses normalized weights within each round so that all seven rounds contributed equally. We estimated Q5–Q1 gaps and ratios, conventional, Wagstaff-normalized, and Erreygers concentration indices, and survey-adjusted Poisson prevalence ratios. A sensitivity analysis was restricted to births in the preceding 12 months. **Results:** The analytic sample included 127,469 most recent live births. Equal-round weighted cesarean prevalence was 37.31% (95% CI, 36.92–37.70), ranging from 17.67% in Q1 to 61.90% in Q5; the absolute gap was 44.23 percentage points (95% CI, 42.92–45.54) and the ratio was 3.50 (95% CI, 3.38–3.64). The conventional, Wagstaff, and Erreygers indices were 0.228, 0.363, and 0.340, respectively. In the primary conditional model, the Q5-versus-Q1 prevalence ratio was 1.94 (95% CI, 1.84–2.04). The gradient remained in the 12-month sensitivity analysis (PR, 2.07; 95% CI, 1.86–2.30) and across marker strata. **Conclusions:** Cesarean delivery among recent live births represented in ENDES was consistently concentrated among wealthier women. These findings document socioeconomic inequality in utilization but do not distinguish clinically appropriate use, overuse, or unmet need.

## 1. Introduction

Cesarean delivery is an essential obstetric intervention when maternal or fetal complications make vaginal birth unsafe. Its public health value depends on prompt availability when clinically indicated, rather than on meeting a fixed population target. The World Health Organization has reported that population-level increases up to approximately 10% are associated with reductions in maternal and neonatal mortality, but it does not recommend a specific national or hospital cesarean rate [1]. Cesarean delivery can involve immediate surgical risks and longer-term maternal and reproductive sequelae [2]. Recent research has also examined uterine scar defects and subsequent gynecologic or obstetric outcomes in relation to uterine closure technique [3,4]. These complications are clinically relevant, but procedure frequency alone cannot determine whether an individual cesarean was appropriate.

Cesarean delivery has increased globally, although the increase is not uniform. International estimates suggest that the global proportion rose from approximately 7% in 1990 to 21% in recent years and could approach 29% by 2030 if current patterns continue [5]. This expansion reflects not only access to obstetric surgery but also changes in clinical practice, health-system organization, financing, maternal preferences, risk perception, and service availability [5,6]. Cesarean delivery is therefore a relevant indicator for examining access, organization of care, and socioeconomic inequality.

Latin America and the Caribbean have among the highest cesarean prevalences worldwide. In 2015, the regional proportion was estimated at approximately 44% [6]. Within-country socioeconomic gradients are also pronounced: births in wealthier households and private facilities tend to have higher cesarean prevalences than births in poorer households [6]. These patterns demonstrate unequal utilization, but without individual clinical indications they cannot establish overuse among wealthier women or unmet obstetric need among poorer women.

In Peru, previous studies have documented high cesarean prevalence and marked geographic and socioeconomic inequalities. An ENDES analysis comparing 2009 and 2018 reported an increase from 21.4% to 34.5%, with higher use among women with greater household wealth, higher education, and urban residence [7]. An analysis of the national birth registry for 2012–2020 likewise found persistently high prevalence, territorial heterogeneity, and higher levels in settings with more favorable socioeconomic indicators [8]. Updated national evidence is needed to quantify inequality after 2018 while addressing the overlapping retrospective windows and survey-specific wealth rankings of successive ENDES rounds.

Accordingly, this study aimed to estimate survey-round-specific rolling prevalence of cesarean delivery by wealth quintile among the most recent live births reported in ENDES rounds 2018–2024; quantify inequality using absolute gaps, relative ratios, and concentration indices; estimate conditional prevalence ratios under sequential adjustment sets; and describe the wealth gradient across strata of selected survey-based obstetric markers. Sensitivity analysis restricted each survey round to births occurring during the preceding 12 months.

## 2. Materials and Methods

### 2.1. Study Design

We conducted an observational, analytical, serial cross-sectional study using secondary microdata from the Peruvian Demographic and Family Health Survey (ENDES) rounds 2018–2024. The unit of analysis was each woman’s most recent reported live birth occurring during the 60 months before the interview, with one birth per woman. This selection avoids within-woman dependence, but it also means that the target of inference is the most recent live births represented in ENDES, rather than all births occurring in Peru. Reporting followed the STROBE recommendations for cross-sectional studies [9]; the checklist is provided in Table S1.

### 2.2. Data Source

ENDES is conducted by Peru’s Instituto Nacional de Estadística e Informática using a probabilistic, stratified, multistage sampling design. The survey provides sampling weights, strata, and primary sampling units for population-representative estimation and design-adjusted uncertainty. Standardized questionnaires and annual microdata are publicly available through the INEI portal [10].

For each survey round, we reconstructed the analytic file from the official REC0111 women’s module, REC21 birth-history module, and REC41 maternal-care module. REC0111 was linked to REC21 by the woman identifier CASEID, and REC21 was linked to REC41 by CASEID and the birth-level indices BIDX and MIDX. Key uniqueness, one most-recent-birth record per woman, and all module linkages were audited before analysis.

### 2.3. Population and Sample

We selected BIDX = 1 and retained births satisfying 0 ≤ V008 − B3 < 60 months, where V008 is the interview date and B3 the child’s date of birth in century-month code. The primary sample required a valid mode of delivery (M17), wealth quintile and score (V190 and V191), a positive sampling weight (V005), and valid cluster and stratum identifiers. All 127,469 temporally eligible most-recent live births matched the REC41 module, and none had missing or out-of-range M17. A prespecified sensitivity analysis applied the shorter interval 0 ≤ V008 − B3 < 12 months.

Eligibility was established before evaluating missingness. Structural blanks corresponding to non-existent birth records were treated as not applicable rather than as item nonresponse. Missing or unknown values in variables used only in secondary models did not determine inclusion in the primary sample; their extent and handling are reported in Table S3.

### 2.4. Variables

The outcome was cesarean delivery, defined from M17 and coded as 1 for cesarean and 0 otherwise. Maternal report of the mode of delivery has shown high agreement with hospital or medical-record data in validation studies, including both short and extended recall periods [11,12]. The main exposure was the survey-specific household wealth quintile V190, categorized from Q1 (poorest) to Q5 (richest). V191, the corresponding continuous asset-based score, was used only to construct socioeconomic ranks within each survey round. Household wealth was measured at interview and was therefore treated as a proxy for socioeconomic position around the time of delivery rather than as a contemporaneously measured exposure.

In brief, V190 represents the household wealth quintile, V191 the continuous wealth score, V008 and B3 the interview and birth dates in century-month code, M17 mode of delivery, M14 antenatal care visits, M15 place of delivery, and M18 maternally perceived fetal size. Maternal age at delivery was calculated as floor ((B3 − V011)/12) and categorized as <20, 20–24, 25–29, 30–34, and ≥35 years. Additional covariates were education (V106), urban or rural residence (V025), region (V024), birth order (BORD), and multiple birth (B0).

Antenatal care visits were categorized as 0–3, 4–5, 6–7, ≥8, and unknown. Place of delivery was classified from the official M15 labels. Home delivery comprised codes 11–12; public facilities codes 21–27; private facilities codes 31–32; and nongovernmental or religious facilities codes 41–42. The primary institutional-birth analysis included codes 21–27, 31–32, and 41–42. Code 96 was labeled “Other” and was excluded from the primary institutional denominator but included in a sensitivity analysis.

We created a descriptive count of selected survey-based obstetric markers available consistently across rounds: maternal age at delivery <20 or ≥35 years, birth order ≥4, multiple birth, and maternally perceived fetal size other than average (M18 codes 1, 2, 4, or 5). The count was categorized as 0, 1, and ≥2 markers. It is not a validated clinical risk score and does not establish an indication or need for cesarean delivery. Equal weighting was used only as a transparent descriptive summary and does not imply equivalent clinical importance. Unknown fetal size was not counted as a positive marker. Sensitivity analyses used a strict fetal-size definition (very large or very small only) and a definition excluding perceived fetal size.

### 2.5. Conceptual Framework

A directed acyclic graph was used to organize the analytic roles of measured variables (Figure S4) [13]. Education and region or area of residence were represented as contextual or socioeconomic determinants associated with household wealth and service access. The selected obstetric markers were treated as pre-delivery characteristics. Antenatal care and place of delivery were treated as healthcare-process variables, while clinical indication and severity, provider and facility factors, and maternal preferences were recognized as incompletely measured. The graph informed sequential conditioning sets; it was not used to claim identification of a causal effect of wealth.

### 2.6. Statistical Analysis

All analyses incorporated the ENDES complex sampling design. Survey-round-specific estimates used the original weight V005/1,000,000. For pooled estimates and regression models, weights were normalized within each round so that all seven rounds contributed equally to the total pooled weight. The pooled estimand is therefore an equal-round average, not an estimate of the total number of births occurring during 2018–2024. Cluster and stratum identifiers were made unique by survey round; strata with a single primary sampling unit were handled using centered variance estimation.

The primary descriptive estimands were survey-round-specific weighted prevalences among the most recent live births occurring 0–59 months before the interview. Because successive rounds contain overlapping retrospective windows, these estimates were interpreted as rolling survey-round estimates rather than calendar-year birth rates. The 0–11-month analysis evaluated robustness to a shorter and more consistent reference period.

The Q5–Q1 absolute gap and Q5/Q1 ratio summarized differences between the extremes of the wealth distribution. The weighted fractional socioeconomic rank was calculated separately within each survey round using V191 and a weighted mid-rank for ties; raw V191 values were not ranked jointly across rounds. The conventional concentration index was calculated as C = 2cov(y,R)/μ, where y denotes cesarean delivery, μ its weighted mean, and R the within-round fractional rank [14,15]. Positive values indicate concentration among wealthier women. Because the feasible bounds of C depend on the mean of a binary outcome, we also reported the Wagstaff-normalized index W = C/(1 − μ) and the Erreygers index E = 4μC [16,17]. Their magnitudes are not directly interchangeable because they embody different relative and absolute inequality properties. Confidence intervals for the indices were obtained from 500 survey-design bootstrap replicates that resampled primary sampling units and recalculated the within-round rank in each replicate.

Associations were estimated using survey-adjusted Poisson regression with a log link, reporting prevalence ratios (PRs) and 95% confidence intervals. This approach was prespecified because cesarean delivery was common and odds ratios would not approximate prevalence ratios [18,19]. Model 0 included wealth quintile only; Model 1 added categorical survey round; Model 2 added maternal age at delivery, birth order, multiple birth, and perceived fetal size; and Model 3, the primary conditional model, further added education, residence, and region. Model 4 exploratorily added antenatal care, retaining unknown visits as an explicit category. Model 5 added detailed place of delivery to Model 3 among institutional births. Models 4 and 5 were interpreted as conditioning on healthcare-process variables, not as causal mediation analyses.

Temporal variation in the wealth gradient was assessed with a categorical wealth-by-round interaction in Model 3. We reported the joint design-adjusted F test and average adjusted predictions standardized over the observed covariate distribution, together with adjusted Q5–Q1 gaps and ratios by round. Multicollinearity was evaluated using generalized variance inflation factors adjusted for the degrees of freedom of categorical terms and the condition index of the model matrix. Trend, interaction, and sensitivity analyses were considered secondary; no formal multiplicity adjustment was applied, and isolated *p* values were interpreted together with effect estimates and confidence intervals. Analyses were performed in R 4.6.0 using the survey package version 4.5; figures were generated from exported analytic results.

### 2.7. Ethical Considerations

The study used only anonymized, publicly available ENDES microdata from INEI [10]. The investigators had no access to personal identifiers and no contact with participants. Institutional ethical review was therefore waived for this secondary analysis, consistent with established considerations for publicly available anonymized data [20].

## 3. Results

### 3.1. Sample Selection

The official annual modules yielded 127,469 most recent live births occurring 0–59 months before interview: 19,824 in 2018, 18,401 in 2019, 15,122 in 2020, 19,181 in 2021, 18,899 in 2022, 18,434 in 2023, and 17,608 in 2024. All eligible REC21 records matched REC41; M17 was missing in 0 records and out of range in 0 records. No further observations were lost because of wealth or survey-design variables. The 0–11-month sensitivity sample included 25,896 births. Figure S1 and Table S3 present the selection and missingness audits.

The earlier denominators of 88,926 and 81,410 originated from a prior harmonized file that did not reproduce the complete official annual linkage. Because the exact mechanism of omission in that file could not be established conclusively, all earlier outputs were discarded, and every analysis reported here was regenerated from the audited REC0111, REC21, and REC41 reconstructions.

### 3.2. Characteristics of the Analytic Population

Table 1 summarizes the analytic population using equal-round pooled weights. Cesarean delivery prevalence was 37.31% (95% CI, 36.92–37.70). Approximately 75.35% of represented births were to women living in urban areas, 36.48% were to women with higher education, and 32.28% were first births. Unknown antenatal care visits accounted for 0.19% of the weighted sample. The selected survey-based obstetric marker count was 0 for 30.15%, 1 for 45.16%, and ≥2 for 24.69%.

### 3.3. Cesarean Delivery Prevalence by Survey Round and Wealth Quintile

The equal-round weighted pooled prevalence was 37.31% (95% CI, 36.92–37.70). Survey-round-specific rolling estimates were 35.60% in 2018, 35.62% in 2019, 36.91% in 2020, 37.51% in 2021, 37.62% in 2022, 38.80% in 2023, and 39.11% in 2024. These values summarize overlapping retrospective windows and are not calendar-year birth rates.

Across the pooled analysis, prevalence increased from 17.67% (95% CI, 17.11–18.23) in Q1 to 31.99% in Q2, 39.95% in Q3, 47.72% in Q4, and 61.90% (95% CI, 60.71–63.09) in Q5. The gradient was present in every survey round (Figure 1 and Table S4).

### 3.4. Socioeconomic Inequality and Wealth-by-Round Interaction

The pooled Q5–Q1 absolute gap was 44.23 percentage points (95% CI, 42.92–45.54), and the Q5/Q1 ratio was 3.50 (95% CI, 3.38–3.64). The conventional concentration index was 0.228 (95% CI, 0.223–0.233), the Wagstaff-normalized index was 0.363 (95% CI, 0.355–0.371), and the Erreygers index was 0.340 (95% CI, 0.331–0.348). All round-specific indices were positive (Table S5 and Figure S2).

The joint wealth-by-round interaction in Model 3 was not statistically significant at the conventional 0.05 level (design-adjusted F = 1.395; numerator df = 24; denominator df = 20,724; *p* = 0.0946). Average adjusted Q5–Q1 gaps ranged from 21.27 to 26.60 percentage points and showed no strictly monotonic temporal pattern (Figure 2 and Table S9).

### 3.5. Prevalence Across Selected Survey-Based Obstetric Marker Strata

A marked wealth gradient was present within every marker stratum (Figure 3 and Table S6). Among births with 0 markers, prevalence was 17.21% in Q1 and 56.57% in Q5. Among births with 1 marker, prevalence was 18.71% in Q1 and 63.47% in Q5. Among births with ≥2 markers, prevalence was 16.73% in Q1 and 66.22% in Q5. The direction of the gradient was unchanged when fetal size was restricted to very large or very small, or when perceived fetal size was removed from the count (Table S10).

### 3.6. Sequential Association Models

In Model 0, the PR comparing Q5 with Q1 was 3.50 (95% CI, 3.38–3.64); after adjustment for survey round in Model 1, it was 3.51 (95% CI, 3.39–3.65). The estimate was 2.69 (95% CI, 2.59–2.80) in Model 2 and 1.94 (95% CI, 1.84–2.04) in Model 3, the primary conditional model. The addition of antenatal care in Model 4 yielded a PR of 1.92 (95% CI, 1.83–2.02). Full estimates are shown in Table 2 and Table S7.

Model 5 was restricted to 119,661 institutional births and included detailed place of delivery. The Q5-versus-Q1 PR was 1.20 (95% CI, 1.15–1.25). Including M15 code 96 (“Other”) in the institutional denominator produced the same rounded estimate. This attenuation was not interpreted as a direct or mediated causal effect because clinical indication, referral severity, and other determinants of place and mode of delivery were incompletely measured.

### 3.7. Sensitivity Analyses, Missingness, and Model Diagnostics

In the 0–11-month sensitivity sample, prevalence was 17.62% in Q1 and 65.84% in Q5, with a gap of 48.21 percentage points (95% CI, 45.46–50.97) and a ratio of 3.74 (95% CI, 3.48–4.01). The Model 3 Q5-versus-Q1 PR was 2.07 (95% CI, 1.86–2.30). The 12-month wealth-by-round interaction was not statistically significant (F = 0.849; numerator df = 24; denominator df = 13,578; *p* = 0.675).

The Model 3 estimate using the original pooled weights was 1.95 (95% CI, 1.85–2.05), and the complete-case antenatal-care model yielded 1.92 (95% CI, 1.82–2.02). Missingness was minimal: M17, wealth, sampling weights, design variables, age at delivery, education, residence, region, birth order, and multiple birth were complete; M14 was unknown for 299 records (0.23% unweighted), M18 for 63 (0.05%), and M15 code 96 represented a valid “Other” place category for 1489 records (1.17%). Multicollinearity diagnostics did not indicate material instability: the largest adjusted generalized variance inflation factor was 1.42 and the largest condition index was 11.57 (Tables S3, S8, and S11).

## 4. Discussion

### 4.1. Main Findings

Cesarean delivery among the most recent live births represented in ENDES was consistently concentrated among wealthier women. The crude prevalence in Q5 was approximately 3.5 times that in Q1, and the absolute gap exceeded 44 percentage points. The gradient remained substantial after conditioning on survey round, measured obstetric characteristics, education, residence, and region, and it was present within every stratum of the selected survey-based marker count. Results were robust to restricting the reference period to 12 months.

The categorical wealth-by-round interaction was not statistically significant, and adjusted gaps did not follow a monotonic pattern. Accordingly, the analysis supports a persistent socioeconomic gradient across survey rounds rather than evidence of a steadily widening or narrowing disparity. Because the main estimates use overlapping retrospective windows, between-round differences should not be interpreted as discrete calendar-year changes.

### 4.2. Comparison with Other Studies and Interpretation of Inequality Measures

The Peruvian pattern is consistent with international evidence of increasing cesarean use and strong within-country socioeconomic gradients [5,6,21]. Previous Peruvian studies likewise found higher cesarean prevalence among wealthier, more educated, and urban women and substantial geographic heterogeneity [7,8]. Our analysis extends that literature by reconstructing official annual survey modules, equalizing the contribution of survey rounds, ranking V191 within each round, quantifying three concentration indices with uncertainty, and assessing short-window sensitivity.

The Q5–Q1 gap and ratio compare the extremes of the wealth distribution, whereas concentration indices use the entire socioeconomic ranking. The conventional index identifies the direction and magnitude of rank-related concentration but has prevalence-dependent bounds for a binary outcome. Wagstaff normalization rescales the conventional index using the outcome mean, whereas the Erreygers correction emphasizes absolute inequality properties [14,15,16,17]. Their numerical values are therefore not directly interchangeable. Their consistently positive direction, rather than their cross-index numerical ordering, supports the conclusion of pro-rich concentration.

The marker-stratified results should not be interpreted as a substitute for the Robson classification or a clinical assessment of need. ENDES lacks previous cesarean status, fetal presentation, onset of labor, gestational age, placental disorders, hypertensive complications, intrapartum fetal status, and other determinants required for clinical classification [22,23,24]. The marker count is, therefore, only a transparent descriptive stratification. Its persistence across alternative definitions supports the robustness of the socioeconomic pattern, not the appropriateness of individual procedures.

### 4.3. Public Health and Temporal Interpretation

The observed gradient is compatible with several nonexclusive mechanisms, including facility mix, financing, referral pathways, provider practice, maternal preferences, geographic accessibility, and timely access to obstetric care. The attenuation after conditioning on place of delivery suggests that care pathways account for part of the conditional association, but it cannot be interpreted as formal mediation because wealth was measured at interview, place and mode of delivery belonged to the same episode, clinical severity was incompletely measured, and home births had no positivity for cesarean delivery.

The findings do not establish that cesareans among wealthier women were unnecessary or that poorer women had unmet surgical need. Those distinctions require individual indications, urgency, labor course, and maternal and neonatal outcomes. Policy should therefore combine equity monitoring with clinical audit and standardized obstetric classification rather than apply a uniform rate target [25]. Evidence-based education, audit and feedback, second-opinion approaches, and organizational changes may be considered where clinically unnecessary use is demonstrated [26,27,28], while timely access must remain protected in populations with barriers to care.

The 2018–2024 period includes the COVID-19 pandemic and changes in service organization and obstetric practice, but the overlapping retrospective windows preclude attributing between-round differences to a particular policy or pandemic phase. The 12-month sensitivity analysis reduced temporal overlap and reproduced the wealth gradient; however, it still does not identify a causal pandemic effect. The most defensible temporal conclusion is the persistence of socioeconomic concentration across ENDES rounds.

### 4.4. Limitations

This study has several limitations. The survey-round estimates summarize overlapping retrospective windows and should not be interpreted as calendar-year cesarean rates. Household wealth was measured at interview rather than at delivery; post-birth changes in assets or household composition may therefore have caused temporal exposure misclassification and, in principle, outcome-related changes in measured socioeconomic position. Mode of delivery was self-reported. Although validation studies generally show high agreement for maternal recall of delivery mode [11,12], Peru-specific validation over the complete five-year recall interval was unavailable.

ENDES does not record individual clinical indication, urgency, labor course, previous cesarean delivery, fetal presentation, or maternal and neonatal outcomes. The selected marker count is not a validated clinical risk score, and residual confounding by clinical, preference, provider, and facility factors remains likely. Restriction to each woman’s most recent reported live birth excludes earlier births, stillbirths, and other pregnancy outcomes; a previous cesarean may also influence subsequent fertility and birth spacing. The results therefore apply to the most recent live births represented in ENDES, not all births in Peru. Missingness in measured covariates was small and explicitly handled, but multiple secondary and sensitivity analyses were conducted without formal multiplicity adjustment; isolated *p* values should be interpreted cautiously.

## 5. Conclusions

Cesarean delivery among the most recent live births represented in ENDES rounds 2018–2024 was persistently concentrated among wealthier women. The gradient remained after sequential adjustment, across selected survey-based obstetric marker strata, and in a 12-month sensitivity analysis. These findings demonstrate socioeconomic inequality in utilization but do not determine whether use in any group was clinically appropriate, unnecessary, or insufficient.

Surveillance should combine survey-based inequality measures with clinical audits, standardized obstetric classifications, and evaluation of public and private care pathways. Future studies linking surveys, birth registries, and clinical records are needed to distinguish indication, timeliness, safety, and maternal or neonatal outcomes more precisely.

## Figures and Tables

**Figure 1:**
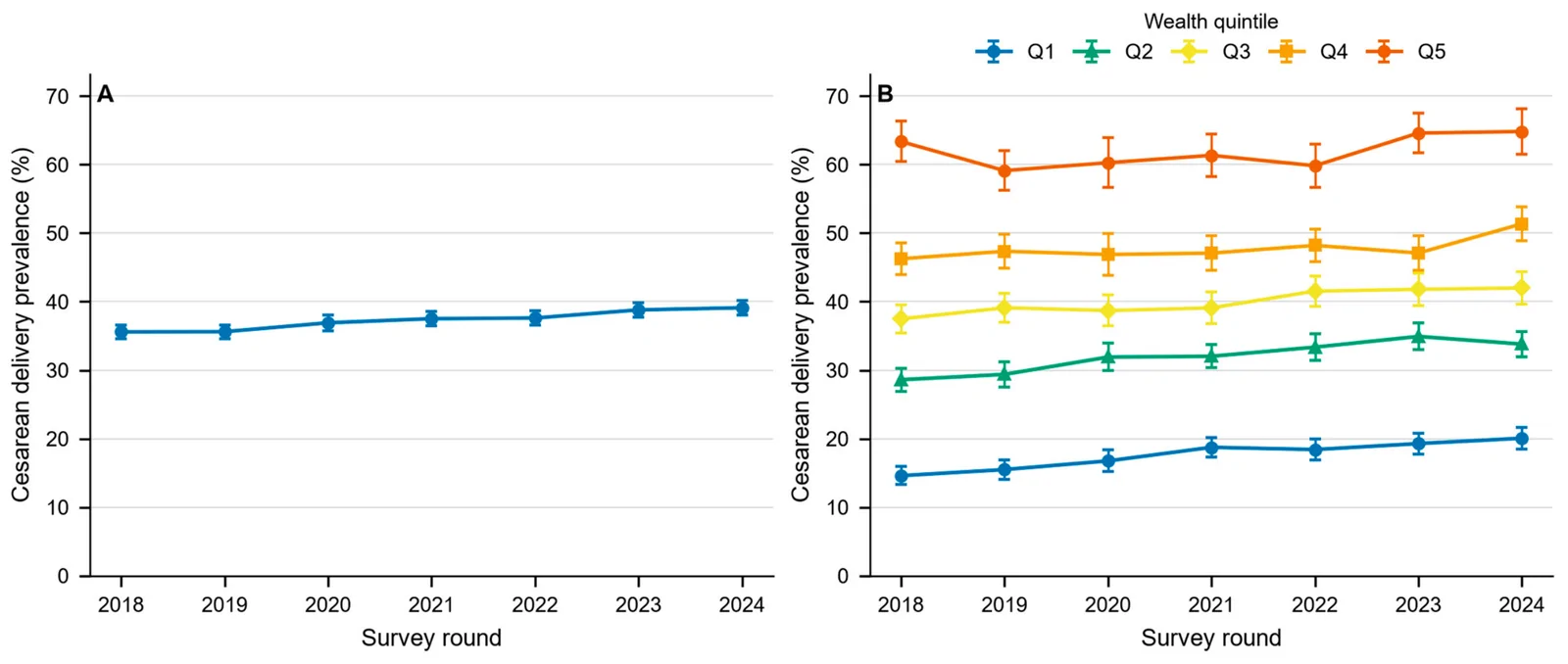
Survey-round-specific weighted prevalence of cesarean delivery overall (**A**) and by wealth quintile (**B**), ENDES Peru 2018–2024. Estimates refer to each woman’s most recent live birth occurring 0–59 months before interview and therefore represent overlapping retrospective windows rather than calendar-year birth rates.

**Figure 2:**
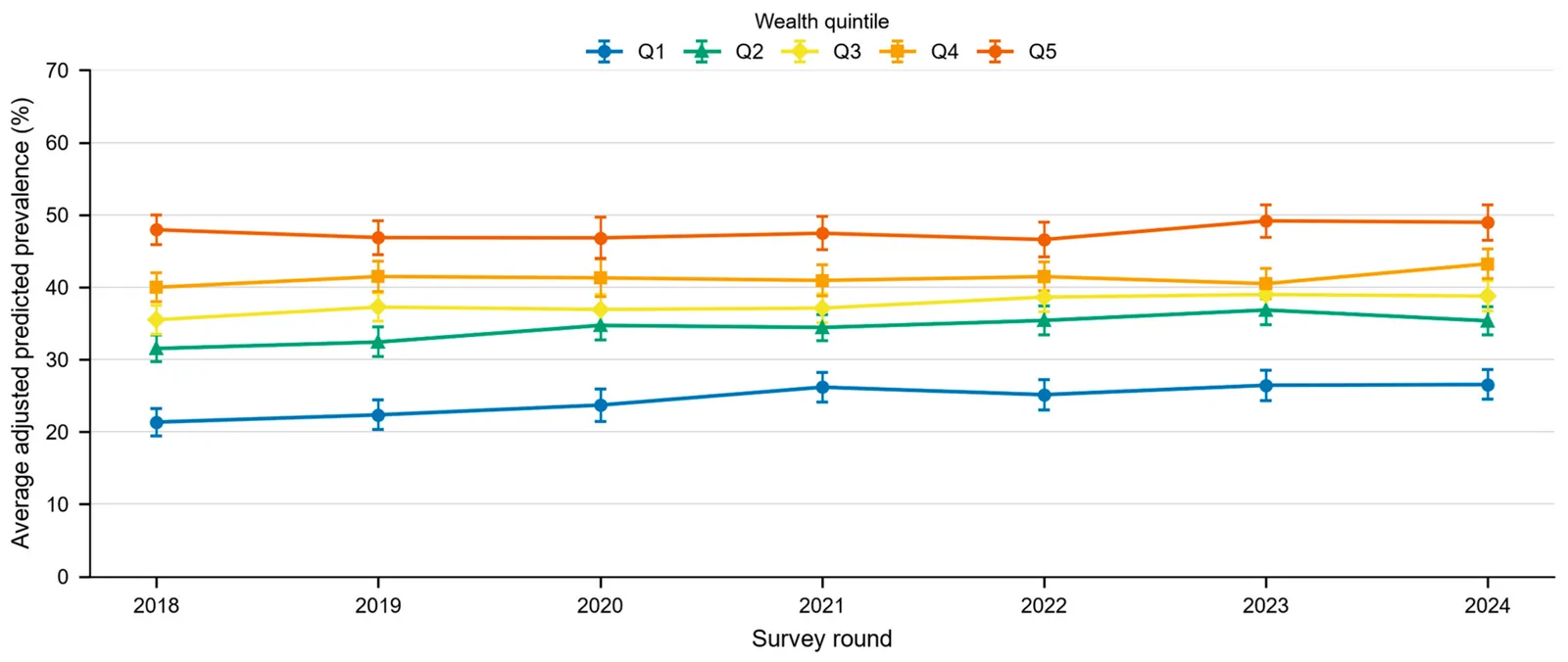
Average adjusted predicted prevalence of cesarean delivery by wealth quintile and survey round from Model 3. Predictions were standardized to the observed covariate distribution; error bars represent 95% confidence intervals.

**Figure 3:**
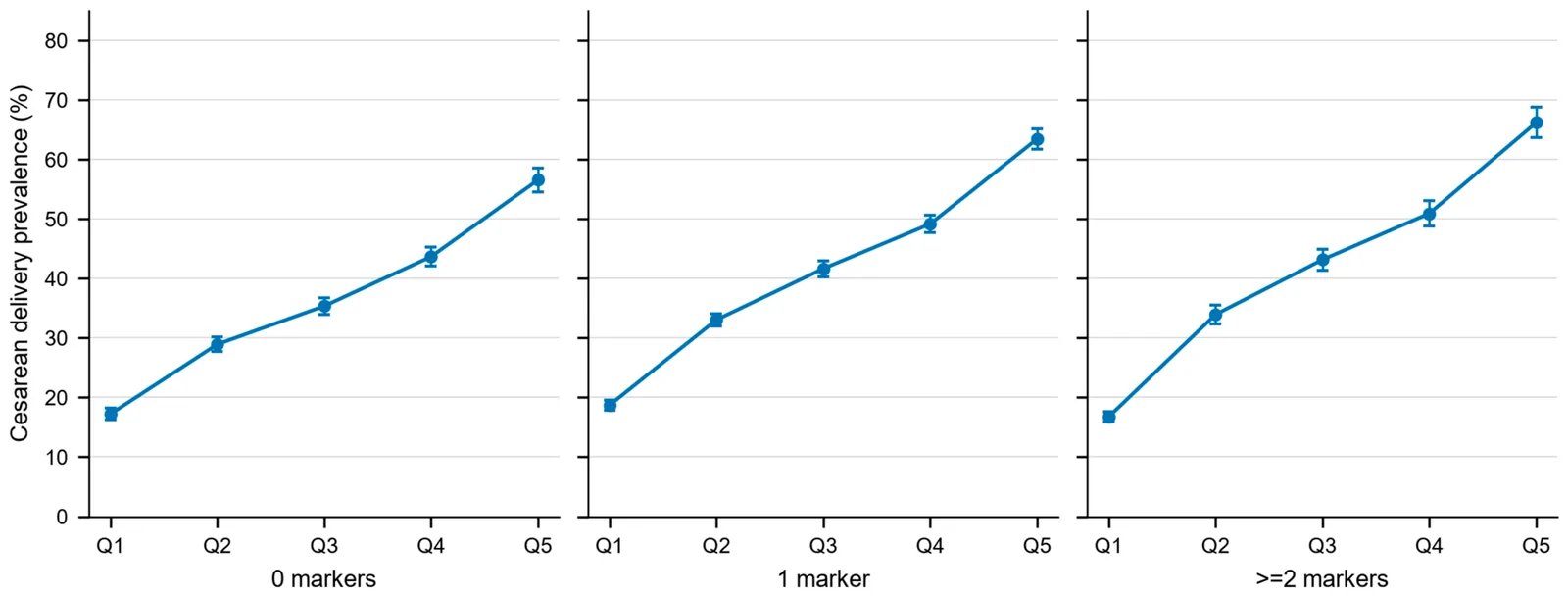
Cesarean delivery prevalence by wealth quintile, stratified by the selected survey-based obstetric marker count, ENDES Peru 2018–2024. Error bars represent 95% confidence intervals.

**Table 1:** Characteristics of the analytical population, ENDES rounds 2018–2024.

Characteristic	Unweighted n	Weighted % (95% CI)
Cesarean delivery: No	83,856	62.69 (62.30–63.08)
Cesarean delivery: Yes	43,613	37.31 (36.92–37.70)
Survey round: 2018	19,824	—
Survey round: 2019	18,401	—
Survey round: 2020	15,122	—
Survey round: 2021	19,181	—
Survey round: 2022	18,899	—
Survey round: 2023	18,434	—
Survey round: 2024	17,608	—
Maternal age at delivery: <20 years	14,609	10.93 (10.70–11.15)
Maternal age at delivery: 20–24 years	27,143	21.07 (20.76–21.38)
Maternal age at delivery: 25–29 years	31,071	24.37 (24.05–24.69)
Maternal age at delivery: 30–34 years	28,187	22.32 (22.00–22.65)
Maternal age at delivery: ≥35 years	26,459	21.31 (21.00–21.62)
Education: No education	1578	1.13 (1.05–1.21)
Education: Primary	21,811	15.92 (15.65–16.19)
Education: Secondary	60,679	46.48 (46.07–46.88)
Education: Higher	43,401	36.48 (36.08–36.87)
Residence: Urban	89,509	75.35 (75.06–75.64)
Residence: Rural	37,960	24.65 (24.36–24.94)
Wealth quintile: Q1	36,357	23.78 (23.45–24.11)
Wealth quintile: Q2	34,098	23.68 (23.31–24.04)
Wealth quintile: Q3	25,614	20.56 (20.22–20.90)
Wealth quintile: Q4	18,809	17.49 (17.17–17.82)
Wealth quintile: Q5	12,591	14.49 (14.16–14.83)
Birth order: First	39,585	32.28 (31.94–32.62)
Birth order: 2–3	65,579	51.64 (51.27–52.01)
Birth order: ≥4	22,305	16.08 (15.81–16.35)
Multiple birth	1131	0.93 (0.86–1.00)
Antenatal care: 0–3 visits	5161	4.31 (4.12–4.49)
Antenatal care: 4–5 visits	9955	8.14 (7.92–8.36)
Antenatal care: 6–7 visits	21,157	16.79 (16.50–17.08)
Antenatal care: ≥8 visits	90,897	70.57 (70.20–70.95)
Antenatal care: Unknown	299	0.19 (0.16–0.23)
Selected survey-based marker count: 0	39,011	30.15 (29.80–30.50)
Selected survey-based marker count: 1	56,453	45.16 (44.79–45.53)
Selected survey-based marker count: ≥2	32,005	24.69 (24.36–25.02)

Pooled weights were normalized within each survey round so that each round contributed equally. Weighted percentages for the survey-round rows are therefore not presented as empirical population proportions. CI: confidence interval.

**Table 2:** Association between wealth quintile and cesarean delivery in sequential survey-adjusted Poisson models.

Wealth Quintile	Model 0	Model 1	Model 2	Model 3	Model 4	Model 5
Q2 versus Q1	1.81 (1.74–1.88)	1.81 (1.75–1.88)	1.69 (1.62–1.75)	1.40 (1.34–1.46)	1.39 (1.33–1.45)	1.07 (1.04–1.11)
Q3 versus Q1	2.26 (2.18–2.35)	2.26 (2.18–2.35)	1.99 (1.91–2.06)	1.53 (1.46–1.60)	1.52 (1.45–1.59)	1.12 (1.08–1.17)
Q4 versus Q1	2.70 (2.60–2.80)	2.71 (2.61–2.81)	2.25 (2.17–2.34)	1.67 (1.59–1.76)	1.67 (1.59–1.75)	1.17 (1.13–1.22)
Q5 versus Q1	3.50 (3.38–3.64)	3.51 (3.39–3.65)	2.69 (2.59–2.80)	1.94 (1.84–2.04)	1.92 (1.83–2.02)	1.20 (1.15–1.25)

Values are prevalence ratios (95% CIs), reference Q1. Model 0: wealth only. Model 1: + survey round. Model 2: + maternal age at delivery, birth order, multiple birth, and perceived fetal size. Model 3: + education, residence, and region (primary conditional model). Model 4: + antenatal care. Model 5: Model 3 + detailed place of delivery, restricted to institutional births (n = 119,661). Models 4 and 5 are exploratory healthcare-process conditioning models, not causal mediation models.

## Data Availability

The microdata used are publicly available through the Instituto Nacional de Estadística e Informática repository: https://proyectos.inei.gob.pe/microdatos/ (accessed on 25 June 2026).

## Supplementary Materials

The following supporting information can be downloaded at https://www.mdpi.com/article/10.3390/healthcare14193263/s1: Table S1: STROBE checklist for cross-sectional observational studies; Table S2: Operational definitions and coding of analytic variables; Table S3: Sample selection, module linkage, and missing-data audit; Table S4: Survey-round-specific cesarean delivery prevalence overall and by wealth quintile; Table S5: Concentration indices by survey round and pooled equal-round estimates; Table S6: Cesarean delivery prevalence by wealth quintile and selected survey-based obstetric marker count; Table S7: Full survey-adjusted Poisson Model 3; Table S8: Sensitivity analyses for Q5 versus Q1; Table S9: Wealth-by-survey-round interaction test and adjusted Q5–Q1 contrasts; Table S10: Sensitivity analyses of the selected survey-based obstetric marker count; Table S11. Multicollinearity diagnostics for Model 3; Figure S1: Analytic sample reconstruction and principal analysis samples; Figure S2: Conventional, Wagstaff-normalized, and Erreygers concentration indices by ENDES survey round. Error bars represent 95% confidence intervals; Figure S3: Cesarean delivery prevalence by wealth quintile and ENDES survey round among most recent live births occurring 0–11 months before interview. Error bars represent 95% confidence intervals; Figure S4: Directed acyclic graph representing the assumed relationships among household wealth, contextual factors, selected survey-based obstetric markers, healthcare-process variables, unmeasured clinical and provider factors, and cesarean delivery. The graph organized sequential adjustment sets and was not used to claim identification of a causal effect.
