# Supplementary material for: Persistent Socioeconomic Concentration of Cesarean Delivery in Peru Across Selected Survey-Based Obstetric Marker Strata: A Serial Cross-Sectional Analysis of ENDES Rounds, 2018–2024
Source: Healthcare (Basel). 2026 Oct 2;14(19):3263. doi: 10.3390/healthcare14193263 (PMC13664888; doi:10.3390/healthcare14193263)

Supplementary Materials

Persistent Socioeconomic Concentration of Cesarean Delivery in Peru Across Selected Survey-Based Obstetric Marker Strata: A Serial Cross-Sectional Analysis of ENDES Rounds, 2018–2024

Víctor Juan Vera-Ponce *, Fiorella E. Zuzunaga-Montoya, Marcos Daniel García-Rodríguez, Witre Omar Padilla and José Celso Paredes Carranza

Supplementary Table S1. STROBE checklist for cross-sectional observational studies.

STROBE item	Domain	Report in the manuscript
1a	Title and abstract	The revised title identifies socioeconomic concentration, survey-based marker strata, and the serial cross-sectional ENDES-round design.
1b	Title and abstract	The abstract reports the rationale, official-module reconstruction, equal-round weighting, main estimates, 12-month sensitivity, and cautious interpretation.
2	Background and rationale	The Introduction describes global, regional, and Peruvian evidence and distinguishes inequality in utilization from clinical appropriateness.
3	Objectives	The final Introduction states objectives for rolling survey-round prevalence, inequality measures, sequential models, marker strata, and a 12-month sensitivity.
4	Study design	Section 2.1 describes an observational, analytical, serial cross-sectional study.
5	Setting	Sections 2.1–2.2 describe ENDES rounds 2018–2024 in Peru and the official annual modules.
6a	Participants	Section 2.3 defines the most recent live birth, the 0–59-month interval, and analytic eligibility.
6b	Participants	No matching was used; not applicable to the cross-sectional survey design.
7	Variables	Section 2.4 and Table S2 define the outcome, exposure, covariates, healthcare-process variables, marker count, and survey-design variables.
8	Data sources and measurement	Sections 2.2–2.4 describe REC0111, REC21, REC41, module linkages, exact DHS variables, maternal recall, and coding.
9	Bias	Methods addresses module linkage, temporal windows, within-woman dependence, and missingness; Discussion addresses exposure timing, recall, selection, and residual confounding.
10	Sample size	All eligible official-module records were analyzed; no a priori sample-size calculation was required for this secondary analysis.
11	Quantitative variables	Sections 2.3–2.4 describe age, months since birth, wealth quintiles and within-round ranks, antenatal visits, and marker-count categorizations.
12a	Statistical methods	Section 2.6 describes survey design, equal-round weights, prevalence estimates, gaps, ratios, concentration indices, bootstrap uncertainty, and modified Poisson models.
12b	Statistical methods	Section 2.6 describes marker stratification, categorical wealth-by-round interaction, average adjusted predictions, and the 12-month sensitivity.
12c	Statistical methods	Section 2.3 and Table S3 describe missingness after eligibility and complete-case sensitivity for antenatal care.
12d	Statistical methods	Section 2.6 describes unique round-specific cluster and stratum identifiers and centered handling of singleton strata.
12e	Statistical methods	Tables S8–S11 report temporal, weight, missing-data, institutional-definition, marker, and multicollinearity sensitivity analyses.
13a	Participants	Results 3.1, Figure S1, and Table S3 report the official reconstruction and final n = 127,469.
13b	Participants	There was no primary recruitment; eligibility and module-linkage results are reported.
13c	Participants	Figure S1 presents the analytic flow.
14a	Descriptive data	Table 1 reports unweighted counts and equal-round weighted percentages with 95% confidence intervals.
14b	Descriptive data	Table S3 reports missing or unknown values globally and their ranges across rounds and wealth quintiles.
14c	Descriptive data	Longitudinal follow-up is not applicable; the analysis is serial cross-sectional.
15	Outcome data	Results, Figures 1–3, and Tables S4–S6 report prevalence overall, by round, wealth, and marker stratum.
16a	Main results	Table 2 and Table S7 report crude and adjusted PRs with 95% CIs; Results reports gaps, ratios, and concentration indices.
16b	Main results	Methods and Table S2 define all category boundaries and reference groups.
16c	Main results	Absolute and relative inequality are reported as prevalence, Q5–Q1 gap, Q5/Q1 ratio, and three concentration indices.

STROBE item	Domain	Report in the manuscript
17	Other analyses	Tables S8–S11 report 12-month, original-weight, complete-case, institutional-definition, marker, interaction, and multicollinearity analyses.
18	Key results	Discussion summarizes persistent pro-rich concentration and the absence of evidence for a monotonic change across rounds.
19	Limitations	Section 4.4 addresses rolling windows, wealth measured at interview, self-report, absent clinical indications, residual confounding, most-recent-live-birth selection, and multiplicity.
20	Interpretation	Discussion interprets estimates as descriptive and conditional associations and explicitly avoids conclusions about overuse, underuse, or causal mediation.
21	Generalizability	Discussion limits inference to most recent live births represented in ENDES and describes relevance for inequality monitoring.
22	Funding	The declarations report funding, ethics, consent, data availability, AI-assisted revision, acknowledgments, and conflicts of interest.

STROBE: Strengthening the Reporting of Observational Studies in Epidemiology.

Supplementary Table S2. Operational definitions and coding of analytic variables.

Variable	DHS source	Definition/coding
Cesarean delivery	M17	1 = cesarean; 0 = non-cesarean. Maternal report.
Wealth quintile	V190	Survey-specific Q1 (poorest) to Q5 (richest).
Continuous wealth score	V191	Used to construct weighted fractional socioeconomic ranks within each survey round; raw values were not pooled across rounds.
Interview and birth dates	V008; B3	Century-month codes; eligible interval $0 \le V008 - B3 < 60$ months; sensitivity 0–11 months.
Most recent live birth	BIDX	BIDX = 1; one birth per woman.
Maternal age at delivery	B3; V011	floor((B3 − V011)/12): <20, 20–24, 25–29, 30–34, ≥35 years.
Education	V106	No education, primary, secondary, higher.
Residence	V025	Urban or rural.
Region	V024	Administrative region categories supplied by ENDES.
Birth order	BORD	First; 2–3; ≥4.
Multiple birth	B0	Singleton or multiple.
Antenatal care visits	M14	0–3, 4–5, 6–7, ≥8, unknown. Unknown retained as explicit category in Model 4; complete-case sensitivity performed.
Place of delivery	M15	Home 11–12; public 21–27; private 31–32; NGO/religious 41–42; Other 96. Main institutional denominator: 21–27, 31–32, 41–42.
Perceived fetal size	M18	Very large, large, average, small, very small, unknown. Unknown was not counted as a positive marker.
Selected survey-based obstetric marker count	Derived	One point each for age <20 or ≥35, birth order ≥4, multiple birth, and M18 codes 1,2,4,5; categories 0, 1, ≥2. Descriptive only, not a validated risk score.
Survey weight	V005	V005/1,000,000 within round. Pooled weights normalized within each round to equal total round contribution.
Cluster and stratum	V021; V022/V023	Made unique by survey round; singleton strata handled with centered variance estimation.

Supplementary Table S3. Sample selection, module linkage, and missing-data audit.

Panel A. Analytic sample flow.

Step	n	Comment
Most recent live birth (BIDX = 1), 0–59 months	127,469	Conceptually eligible base from REC21 + REC0111
REC41 matched by CASEID and BIDX/MIDX	127,469	Unmatched = 0
Valid M17 (0/1)	127,469	Missing = 0; out of range = 0
Positive V005 and valid V190/V191/design identifiers	127,469	Excluded = 0
Final primary analytic sample	127,469	0–59 months
12-month sensitivity sample	25,896	0–11 months
Institutional sample, main definition	119,661	M15 codes 21–27, 31–32, 41–42
Institutional sample including code 96	121,150	Sensitivity only

Panel B. Round-specific primary-sample counts.

Survey round	n
2018	19,824
2019	18,401
2020	15,122
2021	19,181
2022	18,899
2023	18,434
2024	17,608

Panel C. Missing or special values among the 127,469 eligible births.

Variable/status	n	Unweighted %	Weighted %	Handling
M17 mode of delivery	0	0.00	0.00	No exclusion attributable to missing M17.
V190/V191, V005, PSU/stratum	0	0.00	0.00	Complete in the analytic sample.
Maternal age at delivery, education, residence, region, BORD, B0	0	0.00	0.00	Complete in the analytic sample.
M14 unknown antenatal visits	299	0.23	0.20	Explicit unknown category in Model 4; complete-case sensitivity.
M18 unknown perceived fetal size	63	0.05	0.06	Retained as unknown in models; not counted as a positive marker.
M15 code 96, “Other” place	1,489	1.17	1.52	Valid category; excluded from main institutional denominator and included in sensitivity.

Across survey rounds, weighted unknown M14 ranged from 0.05% to 0.34%, unknown M18 from 0.03% to 0.10%, and M15 code 96 from 0.97% to 2.26%. Across wealth quintiles, the corresponding ranges were 0.11%–0.27%, 0.03%–0.13%, and 1.04%–2.33%, respectively.

Supplementary Table S4. Survey-round-specific cesarean delivery prevalence overall and by wealth quintile.

Round	Overall	Q1	Q2	Q3	Q4	Q5
2018	35.60 (34.58–36.61)	14.60 (13.30–15.90)	28.60 (26.90–30.30)	37.49 (35.40–39.57)	46.26 (44.00–48.53)	63.38 (60.43–66.33)
2019	35.62 (34.63–36.60)	15.48 (14.04–16.92)	29.38 (27.54–31.23)	39.10 (37.04–41.17)	47.35 (44.88–49.82)	59.13 (56.25–62.01)
2020	36.91 (35.76–38.06)	16.75 (15.18–18.32)	31.91 (29.91–33.90)	38.70 (36.44–40.96)	46.89 (43.86–49.92)	60.28 (56.67–63.90)
2021	37.51 (36.48–38.54)	18.74 (17.28–20.19)	32.03 (30.33–33.72)	39.09 (36.80–41.38)	47.08 (44.57–49.59)	61.36 (58.29–64.43)
2022	37.62 (36.59–38.64)	18.41 (16.89–19.93)	33.35 (31.41–35.29)	41.54 (39.33–43.75)	48.22 (45.83–50.60)	59.84 (56.70–62.99)
2023	38.80 (37.75–39.85)	19.28 (17.78–20.78)	34.92 (32.96–36.88)	41.80 (39.41–44.19)	47.10 (44.54–49.65)	64.61 (61.68–67.54)
2024	39.11 (38.07–40.15)	20.06 (18.52–21.60)	33.80 (31.92–35.67)	42.01 (39.62–44.40)	51.36 (48.88–53.84)	64.84 (61.51–68.17)

Values are weighted prevalence percentages (95% CIs) using the original weight of each survey round.

Supplementary Table S5. Concentration indices by survey round and pooled equal-round estimates.

Scope	Conventional C	Wagstaff-normalized	Erreygers
2018	0.264 (0.250–0.277)	0.410 (0.388–0.431)	0.376 (0.353–0.396)
2019	0.241 (0.226–0.255)	0.375 (0.353–0.397)	0.344 (0.324–0.364)
2020	0.222 (0.206–0.235)	0.351 (0.324–0.373)	0.327 (0.300–0.349)
2021	0.215 (0.202–0.229)	0.344 (0.322–0.365)	0.323 (0.300–0.342)
2022	0.214 (0.200–0.228)	0.343 (0.323–0.367)	0.322 (0.301–0.347)
2023	0.216 (0.203–0.231)	0.353 (0.332–0.376)	0.336 (0.314–0.358)
2024	0.224 (0.208–0.237)	0.367 (0.343–0.388)	0.350 (0.326–0.370)
Equal-round mean of round-specific indices	0.228 (0.223–0.234)	0.364 (0.355–0.371)	0.340 (0.331–0.348)
Pooled equal-round index	0.228 (0.223–0.233)	0.363 (0.355–0.371)	0.340 (0.331–0.348)

Confidence intervals were obtained from 500 survey-design bootstrap replicates with within-replicate recalculation of socioeconomic ranks.

Supplementary Table S6. Cesarean delivery prevalence by wealth quintile and selected survey-based obstetric marker count.

Marker count	Q1	Q2	Q3	Q4	Q5
0	17.21 (16.25–18.18)	28.94 (27.77–30.12)	35.36 (34.00–36.73)	43.68 (42.09–45.28)	56.57 (54.55–58.59)
1	18.71 (17.86–19.55)	33.05 (32.03–34.06)	41.67 (40.34–42.99)	49.18 (47.73–50.62)	63.47 (61.80–65.13)
≥2 markers	16.73 (15.87–17.59)	33.94 (32.41–35.48)	43.17 (41.40–44.93)	50.94 (48.81–53.07)	66.22 (63.66–68.78)

Values are equal-round weighted prevalence percentages (95% CIs). The count is descriptive and is not a validated clinical risk score.

Supplementary Table S7. Full survey-adjusted Poisson Model 3.

Term/contrast	PR	95% CI	p value
Wealth quintile Q2 vs Q1	1.397	1.338–1.458	<0.001
Wealth quintile Q3 vs Q1	1.526	1.456–1.600	<0.001
Wealth quintile Q4 vs Q1	1.674	1.594–1.759	<0.001
Wealth quintile Q5 vs Q1	1.937	1.840–2.039	<0.001
Survey round 2019	1.022	0.987–1.058	0.229
Survey round 2020	1.039	1.002–1.078	0.040
Survey round 2021	1.051	1.016–1.088	0.004
Survey round 2022	1.061	1.024–1.100	0.001
Survey round 2023	1.086	1.049–1.124	<0.001
Survey round 2024	1.091	1.054–1.129	<0.001
Maternal age 20-24 years vs <20	1.144	1.092–1.199	<0.001
Maternal age 25-29 years vs <20	1.467	1.401–1.538	<0.001
Maternal age 30-34 years vs <20	1.714	1.635–1.798	<0.001
Maternal age >=35 years vs <20	2.005	1.911–2.104	<0.001
Birth order 2-3 vs 1	0.780	0.763–0.798	<0.001
Birth order >=4 vs 1	0.546	0.524–0.569	<0.001
Multiple birth vs singleton	2.292	2.183–2.407	<0.001
Perceived size large vs very large	0.824	0.779–0.872	<0.001
Perceived size average vs very large	0.715	0.676–0.756	<0.001
Perceived size small vs very large	0.805	0.759–0.853	<0.001
Perceived size very small vs very large	1.047	0.978–1.121	0.183
Perceived size unknown vs very large	0.993	0.592–1.664	0.978
Primary education vs no education	1.200	1.039–1.386	0.013
Secondary education vs no education	1.486	1.286–1.717	<0.001
Higher education vs no education	1.708	1.477–1.975	<0.001
Rural residence vs urban	0.889	0.855–0.924	<0.001
Region Ancash	1.032	0.962–1.107	0.382
Region Apurimac	0.926	0.860–0.997	0.043
Region Arequipa	1.066	0.999–1.138	0.055
Region Ayacucho	0.879	0.817–0.945	<0.001
Region Cajamarca	0.853	0.785–0.926	<0.001
Region Callao	0.968	0.905–1.037	0.354
Region Cusco	0.865	0.803–0.933	<0.001
Region Huancavelica	0.808	0.746–0.877	<0.001
Region Huanuco	0.748	0.692–0.809	<0.001
Region Ica	1.018	0.953–1.089	0.591
Region Junin	0.888	0.825–0.955	0.001
Region La Libertad	1.178	1.101–1.260	<0.001
Region Lambayeque	1.174	1.099–1.254	<0.001
Region Lima	1.003	0.943–1.066	0.934
Region Loreto	0.839	0.779–0.904	<0.001
Region Madre de Dios	0.916	0.853–0.984	0.017
Region Moquegua	1.025	0.959–1.096	0.467
Region Pasco	0.781	0.722–0.845	<0.001
Region Piura	1.293	1.208–1.384	<0.001
Region Puno	0.793	0.731–0.859	<0.001
Region San Martin	1.108	1.033–1.187	0.004
Region Tacna	1.084	1.014–1.159	0.018
Region Tumbes	1.303	1.222–1.389	<0.001

	Term/contrast	PR	95% CI	p value
Region Ucayali		0.851	0.790–0.916	<0.001

Model 3 includes wealth quintile, categorical survey round, five maternal-age-at-delivery categories, birth order, multiple birth, perceived fetal size, education, residence, and region. n = 127,469.

Supplementary Table S8. Sensitivity analyses for Q5 versus Q1.

Sensitivity	n	Window	Weight	PR (95% CI)
Model 3 restricted to 0-11 months	25,896	0-11 months	equal_round_weight	2.07 (1.86–2.30)
ANC complete case	127,170	0-59 months	equal_round_weight	1.92 (1.82–2.02)
Original pooled weights	127,469	0-59 months	original_weight	1.95 (1.85–2.05)
Institutional alternative including M15 code 96	121,150	0-59 months	equal_round_weight	1.20 (1.15–1.25)

The 12-month Model 3 interaction test was F = 0.849, numerator df = 24, denominator df = 13,578, p = 0.675.

Supplementary Table S9. Wealth-by-survey-round interaction test and adjusted Q5–Q1 contrasts.

Round	Adjusted Q1 %	Adjusted Q5 %	Adjusted gap, pp (95% CI)	Adjusted ratio (95% CI)
2018	21.33	47.93	26.60 (23.69–29.50)	2.25 (2.03–2.49)
2019	22.34	46.85	24.51 (21.29–27.73)	2.10 (1.88–2.34)
2020	23.69	46.81	23.12 (19.38–26.85)	1.98 (1.76–2.22)
2021	26.16	47.44	21.27 (18.05–24.50)	1.81 (1.65–2.00)
2022	25.13	46.58	21.45 (18.18–24.73)	1.85 (1.68–2.05)
2023	26.43	49.16	22.73 (19.55–25.91)	1.86 (1.69–2.04)
2024	26.52	48.96	22.43 (19.16–25.71)	1.85 (1.68–2.03)

Joint interaction test: design-adjusted F = 1.395; numerator df = 24; denominator df = 20,724; p = 0.0946. Margins are average adjusted predictions standardized over the observed covariate distribution.

Supplementary Table S10. Sensitivity analyses of the selected survey-based obstetric marker count.

Strict fetal-size marker (very large or very small only).

Marker count	Q1	Q2	Q3	Q4	Q5
0	19.18 (18.39–19.97)	31.25 (30.36–32.15)	38.80 (37.71–39.88)	46.19 (44.98–47.41)	58.88 (57.36–60.39)
1	16.16 (15.35–16.96)	32.96 (31.72–34.21)	42.04 (40.50–43.59)	50.19 (48.39–51.98)	66.57 (64.59–68.54)
≥2 markers	16.82 (15.62–18.02)	33.02 (30.71–35.33)	40.56 (37.84–43.29)	50.45 (47.06–53.85)	65.26 (60.99–69.53)

Marker count excluding perceived fetal size.

Marker count	Q1	Q2	Q3	Q4	Q5
0	19.53 (18.75–20.31)	31.97 (31.10–32.85)	39.43 (38.37–40.49)	46.93 (45.74–48.13)	59.64 (58.14–61.14)
1	16.02 (15.22–16.83)	32.66 (31.33–33.99)	41.91 (40.34–43.48)	49.94 (48.14–51.74)	66.77 (64.77–68.76)
≥2 markers	15.66 (14.46–16.86)	29.78 (27.63–31.94)	36.50 (33.61–39.40)	45.87 (42.05–49.70)	59.51 (54.26–64.77)

Supplementary Table S11. Multicollinearity diagnostics for Model 3.

Model term	GVIF	df	GVIF^(1/(2×df))
wealth_f	3.485	4	1.169
source_round_f	1.073	6	1.006
age_cat5	1.696	4	1.068
birth_order_cat	1.851	2	1.166
multiple_birth	1.125	1	1.061
size_cat	1.131	5	1.012
education	1.876	3	1.111
residence	2.013	1	1.419
region	2.282	24	1.017

Maximum condition index = 11.57. These diagnostics did not indicate material coefficient instability.

Supplementary Figure S1. Analytic sample reconstruction and principal analysis samples.

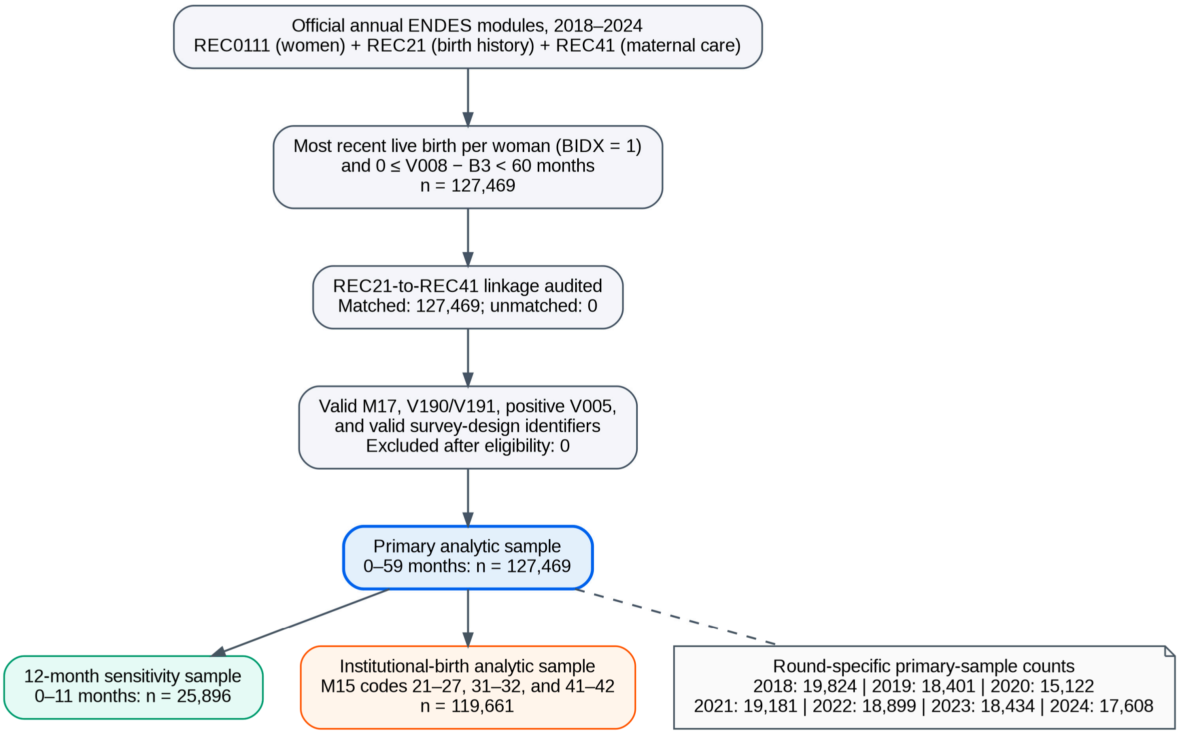

Supplementary Figure S2. Conventional, Wagstaff-normalized, and Erreygers concentration indices by ENDES survey round. Error bars represent 95% confidence intervals.

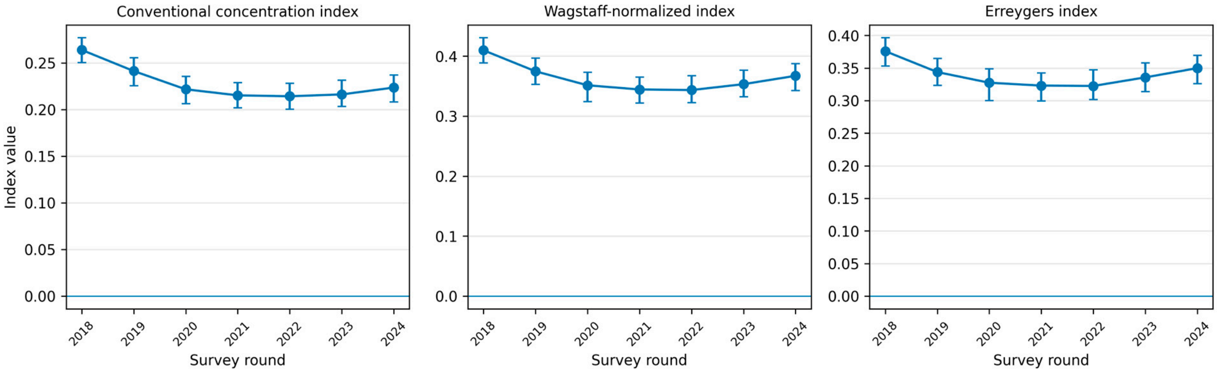

Supplementary Figure S3. Cesarean delivery prevalence by wealth quintile and ENDES survey round among most recent live births occurring 0–11 months before interview. Error bars represent 95% confidence intervals.

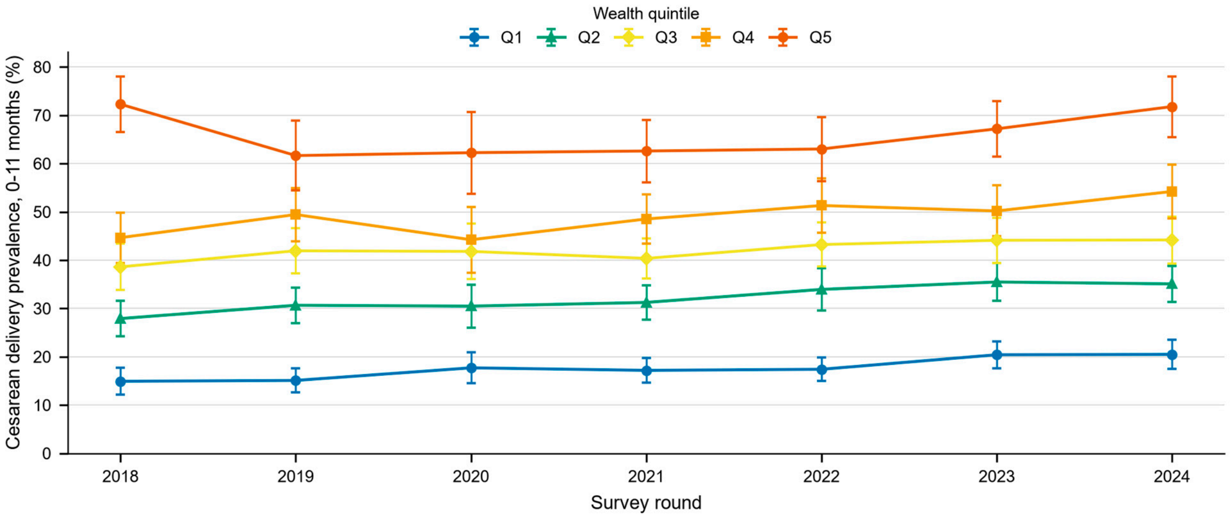

Supplementary Figure S4. Directed acyclic graph representing the assumed relationships among household wealth, contextual factors, selected survey-based obstetric markers, healthcare-process variables, unmeasured clinical and provider factors, and cesarean delivery. The graph organized sequential adjustment sets and was not used to claim identification of a causal effect.

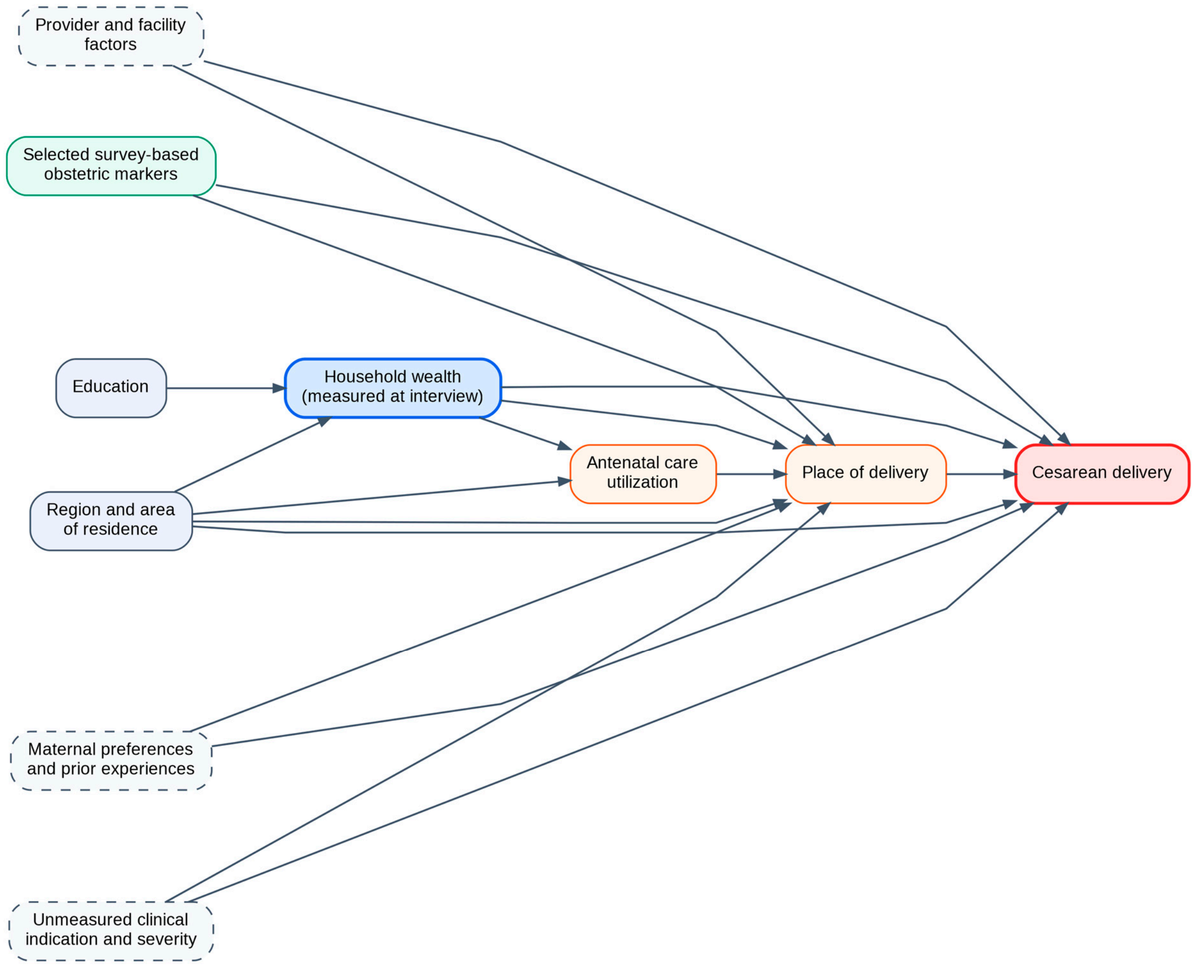

Supplement: Supplementary file 1 [file healthcare-14-03263-s001.zip › healthcare-4430558-supplementary.pdf]
